# Establishment of Epithelial Inflammatory Injury Model Using Intestinal Organoid Cultures

**DOI:** 10.1155/2023/3328655

**Published:** 2023-03-07

**Authors:** Chengfeng Xing, Guili Liang, Xin Yu, Anxing Zhang, Xiang Luo, Yu Liu, Zengli Tang, Bian Wu, Zhengji Song, Danfeng Lan

**Affiliations:** ^1^Department of Gastroenterology, The First People's Hospital of Yunnan Province, The Affiliated Hospital of Kunming University of Science and Technology, No. 157, Jingbi Road, Kunming 650032, Yunnan, China; ^2^Kunming Medical University, No. 1168, West Chunrong Road, Chenggong District, Kunming 650500, Yunnan, China; ^3^Medical School, Kunming University of Science and Technology, Kunming, China; ^4^State Key Laboratory of Primate Biomedical Research, Institute of Primate Translational Medicine, Kunming University of Science and Technology, Kunming 650500, Yunnan, China; ^5^Department of General Surgery II, The First People's Hospital of Yunnan Province, The Affiliated Hospital of Kunming University of Science and Technology, No. 157, Jingbi Road, Kunming 650032, Yunnan, China

## Abstract

Intestinal epithelial dysfunction is critical in the development of inflammatory bowel disease (IBD). However, most cellular experiments related to epithelial barrier studies in IBD have been based on tumor cell line that lack a variety of intestinal epithelial cell types. Thus, intestinal organoids can present the three-dimensional structure and better simulate the physiological structure and function of the intestinal epithelium *in vitro*. Here, the crypts were isolated from the small intestine of mice; with the participation of major cytokines (EGF, Noggin, and R-Spondin 1 included), the intestinal organoids were established at a density of 100 crypts per well, containing intestinal stem cells (ISC), Paneth cells, goblet cells, and intestinal endocrine cells. We found that tumor necrosis factor-alpha (TNF-*α*) could induce the inflammatory response of intestinal organoids, and a dose of 10 ng/mL could maintain stable passaging of organoids for dynamic observation. After stimulation with TNF-*α*, the intestinal organoid cultures showed lower expression of the cell proliferation-related protein identified by monoclonal antibody Ki 67 (*Ki67*), the ISC marker leucine-rich repeat-containing G protein-coupled receptor 5 (*Lgr5*), and the intestinal tight junction proteins occludin (*Ocln*) and claudin-1 (*Cldn1*) while higher expression of the inflammatory cytokine interleukin- (IL-) 15 and the chemokines C-X-C motif ligand 2 (*Cxcl2*) and *Cxcl10* significantly. In this study, we successfully established an epithelial inflammatory injury model of intestinal organoids, which provides an effective *in vitro* model for studying the pathogenesis and treatment of IBD.

## 1. Introduction

The intestinal epithelial cell layer is characterized by an enormous self-renewing capacity (every 4–5 days). The intestinal stem cells at the crypt bottom give rise to new stem cells. Along the crypt-villus axis, new stem cells differentiate into various types of intestinal epithelial cells. Finally, the differentiated epithelial cells are shed into the gut lumen [1]. A strict balance between cell proliferation at the crypt bottom and cell death at the villus tip must be preserved, which is indispensable for maintaining intestinal homeostasis [2]. Abnormal proliferation may increase cell death and lead to separation of the epithelial barrier, followed by infiltration of bacteria and development of intestinal inflammation, which can lead to inflammatory bowel disease (IBD) [3]. IBD is a chronic inflammatory disorder of the intestine that includes Crohn's disease and ulcerative colitis, which can cause lifelong inflammatory disorders and severely impair the quality of life of patients [4]. This disease is thought to primarily occur in European and American countries several decades ago, but its incidence has been increasing in developing countries since the 21st century [5]. Although the etiopathogenesis of IBD is not completely understood, the consensus is that in genetically predisposed individuals, a combination of immune disorders with intrinsic (epithelial barrier function) and extrinsic (microbiota) factors leads to the development of chronic inflammation [6].

The intestinal epithelium is an important factor in the pathogenesis of IBD. Studies on the intestinal epithelium and its interaction with intestinal bacteria and the immune system are helpful in revealing the pathogenesis of IBD [7]. Chronic mucosal inflammation in IBD patients produced high levels of proinflammatory cytokines such as tumor necrosis factor-alpha (TNF-*α*), interleukin- (IL-) 6, and IL-1 [8]. At present, the *in vitro* experimental model of intestinal epithelial studies associated with IBD mainly comprises intestinal epithelial cell (IEC) monolayers (such as Caco-2 cell lines) derived from tumor cells, which is not sufficiently a representative of the intestinal epithelial barrier *in vivo*. A single cell type cannot reveal the interaction between various IECs. Moreover, the inflammatory model of IEC monolayers treated with chemical reagents, inflammatory cytokines, and irradiation is not suitable for dynamic detection of the cell growth trajectory. Therefore, novel models that focus on intestinal epithelial biology are necessary in the context of IBD.

In recent years, organoid technology has become a powerful tool for studying diseases and their development *in vitro*. One major feature of organoid cultures is the presence of both stem cells and differentiated lineages, which can self-organize into multicellular three-dimensional (3D) structures that simulate critical histological and functional aspects of *in vivo* tissue. Organoids have been widely used in drug screening, disease modeling, and host-microbe interaction studies [9–11]. The 3D culture system of intestinal organoids derived from *Lgr5*^+^ intestinal stem cells (ISCs) provides a feasible and credible model for the study of the intestinal epithelium in IBD. To better simulate the inflammatory environment of intestinal epithelium, we tried to establish the epithelial inflammatory injury model using intestinal organoid cultures induced by proinflammatory cytokines (including IL-1*β*, IL-6, and TNF-*α*).

## 2. Methods

### 2.1. Mice

Six- to twelve-week-old C57BL/6 mice were obtained from the Animal Center of Kunming University of Science and Technology. The small intestinal mucosae of the mice were used for crypt isolation and intestinal organoid culture. All experiments were performed with the approval of the Animal Care and Use Committee of Kunming University of Science and Technology and in accordance with the institutional regulations.

### 2.2. Crypt Isolation and Intestinal Organoid Culture

Murine intestinal crypts were isolated and cultured as previously described [12, 13]. Briefly, the isolated small intestine was opened longitudinally by washing with cold Dulbecco's phosphate-buffered saline without Ca^2+^/Mg^2+^ (DPBS). The tissue was cut into 1–5 mm pieces and continually washed at least 10 times with cold DPBS until the supernatant became clear. Tissue fragments were incubated in 2.5 mM EDTA (Invitrogen, USA) with DPBS for 30 min on ice and then vigorously suspended using a 25 mL pipette with cold DPBS. This fraction was passed through a 70 mm cell strainer to remove residual villous material and further enrich the crypts. The filtered supernatant was centrifuged at 300 × g for 5 min to collect the crypts. Approximately 100 crypts were embedded in 10 *μ*L Matrigel (growth factor reduced, ABW, China) in 96-well plates and incubated for 20 min for the Matrigel to solidify. Then, 100 *μ*L basal culture medium containing advanced DMEM/F12 (Gibco, USA), 10 mM HEPES (Invitrogen, USA), 1x GlutaMAX (Thermo Scientific, USA), 1% penicillin/streptomycin (Invitrogen, USA), 1x N2 supplement (Invitrogen, USA), 1x B27 supplement (Invitrogen, USA), and 1 mM N-acetylcysteine (Sigma-Aldrich, USA) was added. For ENR (EGF/Noggin/R-Spondin 1) organoids, a basal culture medium containing 50 ng/mL human EGF (STEMCELL, Canada), 100 ng/mL murine Noggin (Invitrogen, USA), and 500 ng/mL human R-Spondin 1 (Invitrogen, USA) was used. The organoid area was calculated using the ImageJ digital processing software [14].

### 2.3. Treatments

The experiments were performed using postpassage intestinal organoids. At the beginning of the passage, the intestinal organoids in the experimental groups were treated with proinflammatory cytokines IL-1*β*, IL-6, or TNF-*α* (PeproTech, USA) at concentrations of 10 or 40 ng/mL and continuously observed for 3 days.

### 2.4. RNA Extraction and Reverse Transcription

The intestinal organoids were collected and cultured for 3 days. The cells were rinsed with cold DPBS once, and 1 mL TRIzol™ reagent (Invitrogen, USA) was added after centrifugation. Total RNA was extracted according to the manufacturer's instructions. A 200 ng RNA template was used to generate cDNA using the PrimeScript™ RT reagent kit with gDNA Eraser (Perfect Real Time) (Takara, China).

### 2.5. Real-Time Quantitative PCR Analysis

Real-time quantitative PCR (qRT-PCR) was performed on a Bio-Rad CFX Connect Real-Time PCR Detection System using PerfectStart® Green qPCR SuperMix (TransGen Biotech, China) according to the manufacturer's instructions. All experiments included a standard curve, and all samples were analyzed in duplicate and expressed as relative amounts (2^−*ΔΔ*Ct^). The relative expression of targeted genes was normalized to that of glyceraldehyde-3-phosphate dehydrogenase (*Gapdh*). The primer sequences used for qRT-PCR are listed in Supplementary Table 1.

### 2.6. Immunofluorescence Staining

**Figure 1:**
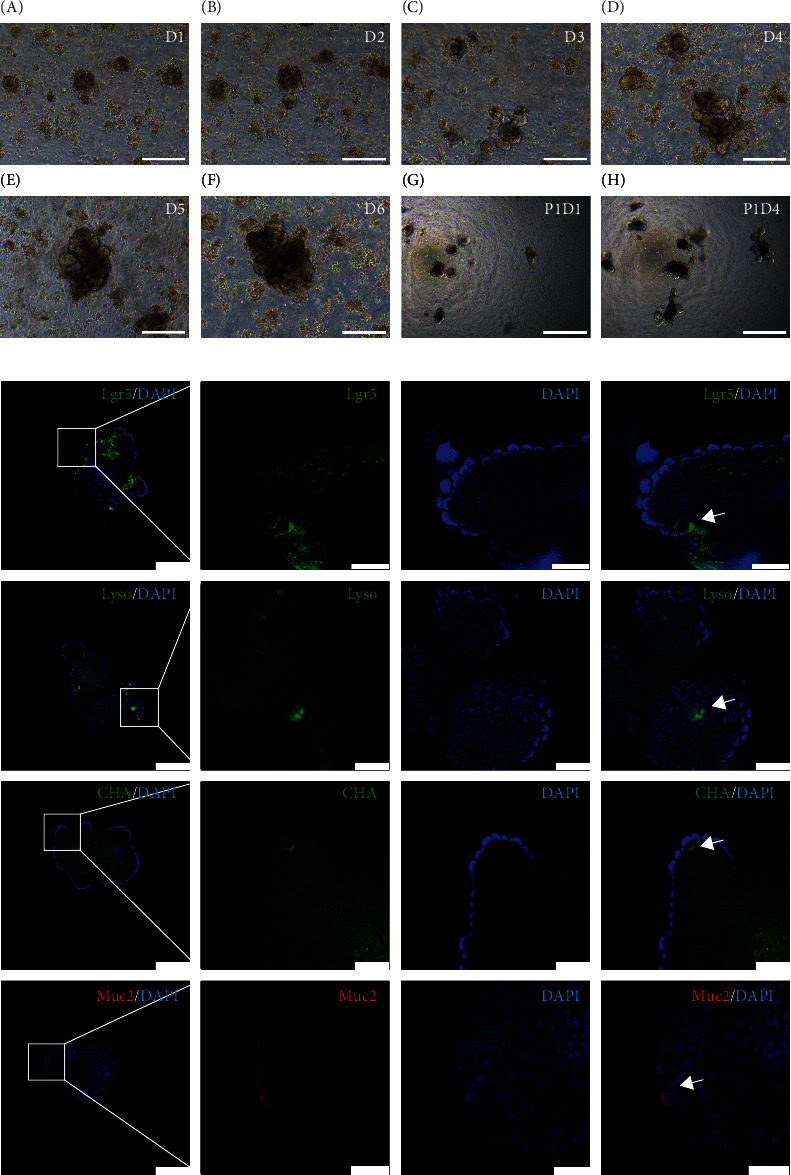
The growth trajectory and epithelial cell type identification of intestinal organoids. (a, A–F) Primary culture of intestinal organoids, images taken at day 1, day 2, day 3, day 4, day 5, and day 6, respectively. Scale bars = 200 *μ*m. (a, G–H) Passage culture of intestinal organoids, images taken at day 1 and day 4. Scale bars = 500 *μ*m. (b) The cell type diversity of intestinal organoids identified by immunofluorescence staining. Confocal image for Lgr5 (green, intestinal stem cell), lysozyme (Lyso, green, Paneth cell), chromogranin A (CHA, green, intestinal endocrine cell), and Muc2 (red, goblet cell). DAPI (blue) for nucleus. The arrows pointed to the positive cells. Scale bars = 100 *μ*m (first column). Scale bars = 20 *μ*m (second, third, and fourth columns).

**Figure 2:**
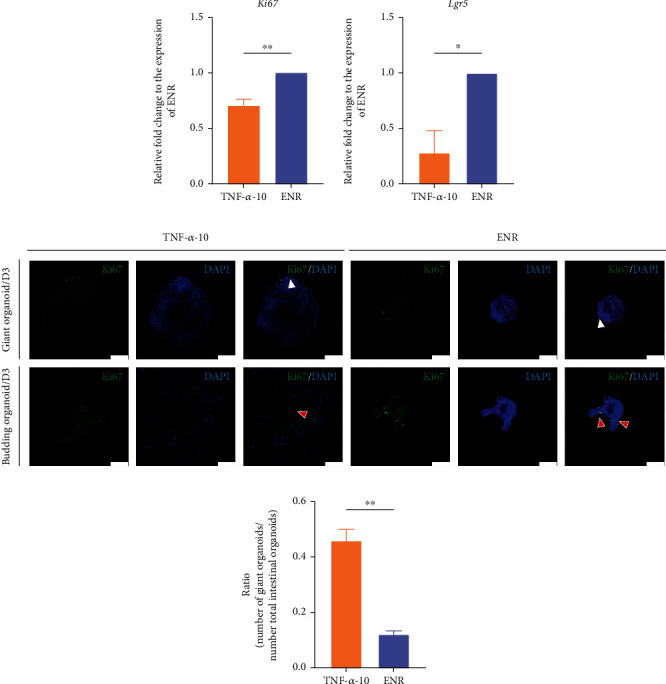
Proliferation ability assay of intestinal organoid with TNF-*α* treatment. (a) qRT-PCR analyses of proliferation marker Ki67 and ISC marker Lgr5 expression in organoids cultured under the indicated conditions (*n* = 1 well). *P* values were determined by two-sided unpaired Student's *t*-test. *Gapdh* was used as a control to normalize the expression of target genes. ^∗^*P* < 0.05; ^∗∗^*P* < 0.01; ^∗∗∗^*P* < 0.001. (b) Immunofluorescence staining of Ki67 (green) of intestinal organoids after 3 days of culture in groups TNF-*α*-10 (first column) and ENR (second column). Images of typical giant organoids (first row) and budding organoids (second row). Blue stain (DAPI) indicates the nucleus. Scale bars = 100 *μ*m. White arrows pointed to the *Ki67*^+^ cells, and red arrows pointed to typical buddings. (c) The ratio of the number of giant organoids and the total number of intestinal organoids (giant organoids plus budding organoids). DAPI nucleus labeling was used in morphological differences of organoid cultures to visualize the budding organoids and giant organoids. ^∗^*P* < 0.05; ^∗∗^*P* < 0.01; ^∗∗∗^*P* < 0.001. The experiments (a, c) were independently repeated at least three times with similar results.

**Figure 3:**
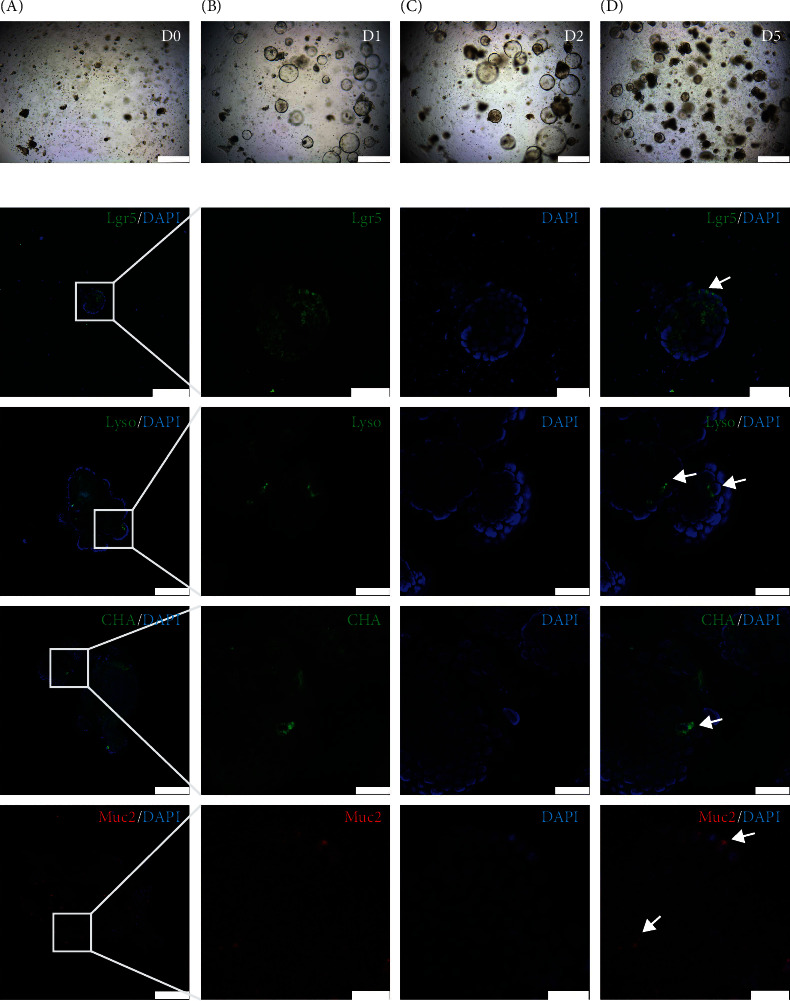
The growth trajectory and epithelial cell type identification of intestinal organoids with epithelial inflammatory injury. (a, A–D) Images taken at day 0, day 1, day 2, and day 5, respectively. Scale bars = 500 *μ*m. (b) Cell type diversity of intestinal organoids with epithelial inflammatory injury identified by immunofluorescence staining. Confocal image for Lgr5 (green, intestinal stem cell), lysozyme (Lyso, green, Paneth cell), chromogranin A (CHA, green, intestinal endocrine cell), and Muc2 (red, goblet cell). DAPI (blue) for nucleus. The arrows pointed to the positive cells. Scale bars = 100 *μ*m (first column). Scale bars = 20 *μ*m (second, third, and fourth columns).

**Figure 4:**
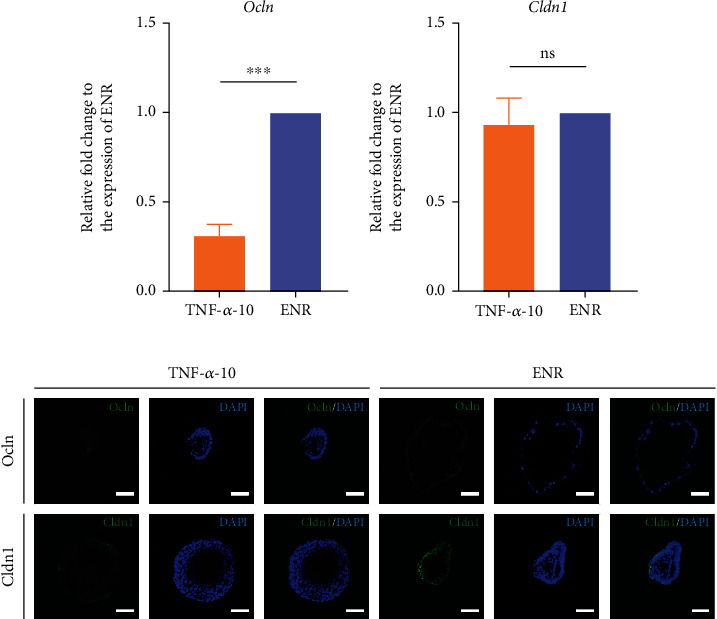
TJP expressions of intestinal organoid with TNF-*α* treatment. qRT-PCR analyses of Ocln (a) and Cldn1 (b) mRNA in organoids cultured under the indicated conditions (*n* = 1 well). *P* values were determined by two-sided unpaired Student's *t*-test. *Gapdh* was used as a control to normalize the expression of target genes. ^∗^*P* < 0.05; ^∗∗^*P* < 0.01; ^∗∗∗^*P* < 0.001. (c) Immunofluorescence staining of representative TJPs of intestinal organoids, including Ocln (first row, green) and Cldn1 (second row, green). Scale bars = 50 *μ*m. The experiments were independently repeated at least three times with similar results. Ocln: occludin; Cldn1: claudin-1.

Each 96-well plate was centrifuged at 300 × g for 5 before fixation. The organoids were fixed in 4% PFA (Biosharp, China) for 45 min at room temperature. (a) Organoids (suspended in the PFA) were moved to the slide and dried the slide slightly, which facilitates the organoids to adhere tightly to the slide (Figures 1(b), 2(b), and 3(b); in this way, the morphology and size of organoids could be well displayed) [15]; (b) organoids were mounted in Tissue-Tek® O.C.T. Compound (Sakura, USA) in -20°C refrigerator and sectioned into 10 *μ*m slices by a cryostat (CM1950, Leica Biosystems) (Figure 4(c); in this way, the proteins on cell membrane could be well displayed, such as tight junction proteins) [16]. Organoids (on the slide) were washed and permeabilized with 0.4% Triton X-100 in PBS (Sigma-Aldrich, USA) for 10 min and blocked with 3% bovine serum albumin (Sigma-Aldrich, USA) in PBS for 1 h. Primary antibodies Ki67 1 : 400 (Abcam, UK), Lgr5 1 : 100 (Affinity, China), Muc2 1 : 100 (Abcam, UK), lysozyme (Lyso) 1 : 100 (Abcam, UK), chromogranin A1 (CHA) 1 : 100 (Abcam, UK), occludin 1 : 100 (Affinity, China), and claudin-1 1 : 100 (Affinity, China) were used and incubated at 4°C overnight. The primary antibodies were then removed, followed by incubation with goat anti-rabbit IgG (H+L) cross-adsorbed secondary antibody 1 : 500 (Invitrogen, USA) and goat anti-mouse IgG (H + L) secondary antibody 1 : 500 (Invitrogen, USA) for 1 h at room temperature in the dark. Nuclei were stained with DAPI (Solarbio, China). Images of organoids were taken by confocal microscopy using a Leica SP8. ImageJ software was used to process and analyze images. Approximately ten images at 10x on slides were analyzed for each condition. The experiments were independently repeated at least three times.

### 2.7. Statistical Analysis

The experiments were performed at least three times. Data are expressed as the mean ± SEM. The statistical significance of differences between mean values was assessed using unpaired two-tailed Student's *t*-tests and one-way ANOVA. All statistical analyses were performed using GraphPad Prism software (version 9.0.0). Statistical significance was defined as a *P* value < 0.05.

## 3. Results

### 3.1. Establishment of Intestinal Organoid Cultures

Mouse crypt preparations were suspended in Matrigel. The crypt supernatant and Matrigel were mixed at a ratio of 1 : 1. Small intestinal organoids were cultured in a 96-well plate containing 10 *μ*L mixed Matrigel and approximately 100 crypts. The growth trajectory and passage culture of the intestinal organoids are shown in Figure 1(a). Single crypts underwent multiple crypt fission events and simultaneously generated villus-like epithelial domains containing differentiated cell types such as Paneth cells (lysozyme), intestinal stem cells (Lgr5), goblet cells (Muc2), and enteroendocrine cells (chromogranin A1, CHA) (Figure 1(b)). The organoid culture was maintained and passaged once before treatment with inflammatory cytokines.

### 3.2. The Growth of Intestinal Organoids Slowed Down Obviously after Treatment with 10 ng/mL TNF-*α*

**Figure 5:**
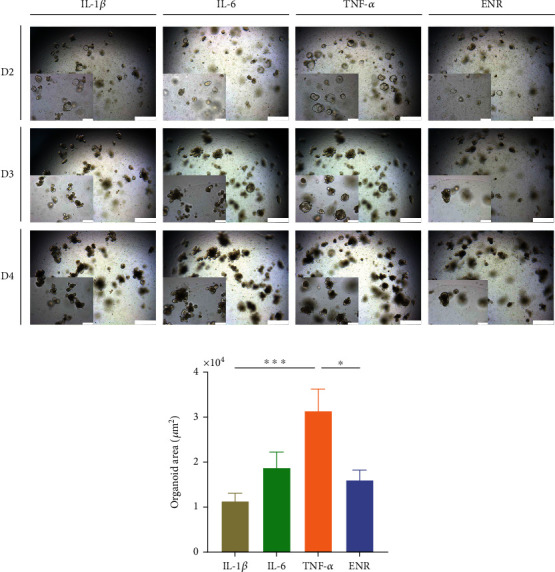
Intestinal organoids with different proinflammatory cytokine treatments. (a) Images of intestinal organoids with different proinflammatory cytokine treatments. IL-1*β* (first column), IL-6 (second column), TNF-*α* (dose: 10 ng/mL; third column), and untreated control (ENR, fourth column). Images were taken continuously for 4 days of culture: day 2 (first row), day 3 (second row), and day 4 (third row). Scale bars = 500 *μ*m. Bottom left corners displayed a zoomed-in intestinal organoids from each image. Scale bars = 200 *μ*m. (b) The organoid area with different proinflammatory cytokine treatments. *P* values were determined by one-way ANOVA. ^∗^*P* < 0.05; ^∗∗^*P* < 0.01; ^∗∗∗^*P* < 0.001. The experiments were independently repeated at least three times with similar results. ENR: EGF/Noggin/R-Spondin 1.

**Figure 6:**
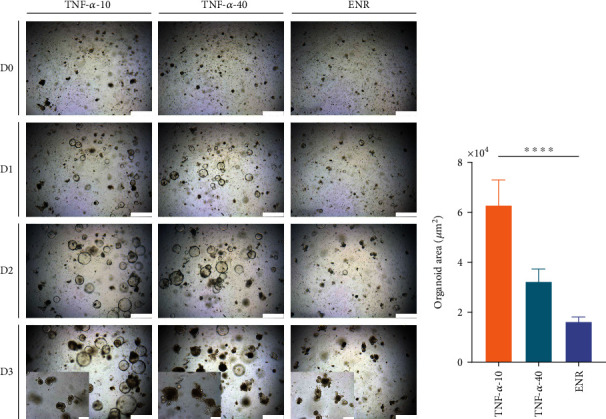
Intestinal organoids with different concentrations of TNF-*α* treatment. (a) Images of intestinal organoids with different concentrations of TNF-*α* treatment. TNF-*α*-10 (dose: 10 ng/mL; first column), TNF-*α*-40 (dose: 40 ng/mL; second column), and untreated control (ENR, third column). Images taken continuously for 3 days of culture: day 0 (first row), day 1 (second row), day 2 (third row), and day 3 (fourth row). Scale bars = 500 *μ*m. Bottom left corners displayed a zoomed-in intestinal organoids. Scale bars = 200 *μ*m. (b) The organoid area with different concentrations of TNF-*α* treatment. *P* values were determined by one-way ANOVA. ^∗^*P* < 0.05; ^∗∗^*P* < 0.01; ^∗∗∗^*P* < 0.001. The experiments were independently repeated at least three times with similar results.

The intestinal organoid was treated with proinflammatory cytokines IL-1*β*, IL-6, and TNF-*α* at a 10 ng/mL dose. The structure of intestinal organoids treated with TNF-*α* was larger, which was significantly different from the other groups (Figure 5). We then focused on TNF-*α* and set different treatment doses of 10 ng/mL and 40 ng/mL, respectively (TNF-*α*-10, TNF-*α*-40, and ENR groups). On day 1, the intestinal organoids in the TNF-*α*-10 and TNF-*α*-40 groups showed morphological changes compared to those in the ENR group, specifically giant organoids. On day 2, fewer giant organoids began to appear as budding structures and increased in size (mainly the lumen diameter). At this time, fewer dead cells were observed in the lumen. On day 3, intestinal organoids treated with 40 ng/mL TNF-*α* showed more budding than on day 2 but could not be cultured stably because of the numerous dead cells in the lumen. Therefore, we chose 10 ng/mL of TNF-*α* for the next experiment. The growth trajectory of intestinal organoids showed that the growth of intestinal organoids treated with 10 ng/mL TNF-*α* lagged behind that of the ENR group (Figure 6).

### 3.3. Intestinal Organoid Proliferation Decreased after Treatment with TNF-*α*

Giant organoids were dominant in the intestinal organoids of the TNF-*α*-10 group (Figure 6). On day 3, giant organoids did not appear to have many budding structures in the TNF-*α*-10 group, whereas numerous budding structures appeared in the ENR group (Figures 5 and 6). Based on immunofluorescence staining, both giant organoids and budding structures only expressed *Ki67* in localized sites (Figure 2(b)). *Ki67*^+^ cells in giant organoids proliferated and differentiated into budding structures, which meant that the giant organoids obviously grew slower than budding organoids. Meanwhile, the proportion of giant organoids was higher in the TNF-*α*-10 group than in the ENR group (Figure 2(c)), which meant that the overall growth rate lagged behind that of normal intestinal organoids.

After 3 days of treatment with TNF-*α* (dose: 10 ng/mL), the intestinal organoids were collected, and the expressions of *Ki67* and *Lgr5* were estimated using qRT-PCR. Similarly, the results showed that their expression significantly decreased after stimulation with 10 ng/mL TNF-*α* (*P* < 0.05) (Figure 2(a)). The morphological observations (especially the proportion of giant organoids) and qRT-PCR data suggest a decreased proliferation ability of intestinal organoids treated with 10 ng/mL TNF-*α*.

### 3.4. TNF-*α* Can Induce an Inflammatory Phenotype in Intestinal Organoids

**Figure 7:**
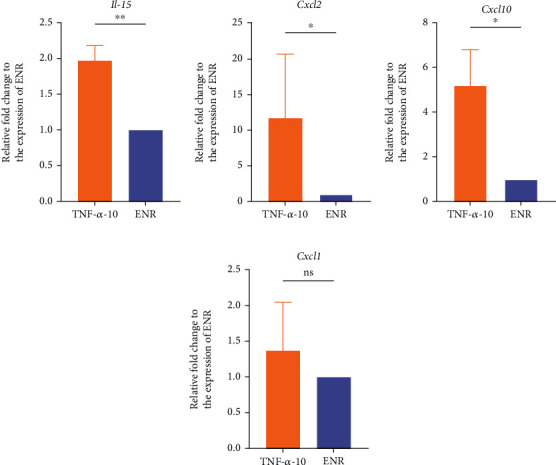
qRT-PCR analyses for inflammatory cytokine and chemokine gene expression. qRT-PCR analyses of expression of the cytokine IL-15 (a), chemokines Cxcl2 (b), Cxcl10(c), and Cxcl1 (d) in organoids cultured under the indicated conditions (*n* = 1 well). *P* values were determined by two-sided unpaired Student's *t*-test. *Gapdh* was used as a control to normalize the expression of target genes. ^∗^*P* < 0.05; ^∗∗^*P* < 0.01; ^∗∗∗^*P* < 0.001. The experiments were independently repeated at least three times with similar results.

Intestinal organoids were collected after 3 days of treatment with TNF-*α*, and the expression of related signaling molecules downstream of the TNF-*α* pathway was detected. The qRT-PCR results showed that the levels of the inflammatory cytokines *Il-15*, *Cxcl2*, and *Cxcl10* were significantly increased (*P* < 0.05) in the TNF-*α*-10 group (Figures 7(a)–7(c)). There was no significant difference in the expression of *Cxcl1* between the TNF-*α*-10 and ENR groups (Figure 7(d)). Tight junction proteins (TJPs) between the intestinal epithelium were measured using qRT-PCR and immunofluorescence staining. The qRT-PCR results showed that the occludin mRNA was significantly decreased compared with that in the ENR group (Figure 4(a)), but the claudin-1 mRNA did not change significantly (Figure 4(b)). We examined the protein expression of occludin and claudin-1; they were all downregulated after TNF-*α* treatment for 3 days; meanwhile, there was a discontinuity in the presence of tight junction proteins in the TNF-*α*-10 group (Figure 4(c)). These data suggest that TNF-*α* can induce an inflammatory phenotype in the intestinal organoids.

### 3.5. Cell Composition Is Identical between Inflamed Intestinal Organoids and Controls

To reduce the interference with intestinal debris that could not be fully filtered in the primary culture, the intestinal organoids of the primary culture were passaged once for subsequent experiments. The growth trajectory images showed that the growth of intestinal organoids treated with TNF-*α* lagged behind that of the controls for more than 3 days (Figure 3(a)). The intestinal organoids with an inflammatory phenotype eventually differentiated into buddings, and immunofluorescence analysis demonstrated that they also contained all cell types of the intestinal epithelium, such as Paneth cells (Lyso), intestinal stem cells (Lgr5), goblet cells (Muc2), and enteroendocrine cells (CHA) (Figure 3(b)). These data suggest that the cell composition is identical between the control and intestinal organoids with an inflammatory phenotype.

## 4. Discussion

The vital functions of the intestines, including digestion, absorption, and acting as a surface barrier, are performed by the intestinal epithelium composed of various differentiated cells and ISCs. The occurrence of many intestinal diseases, including IBD, is related to intestinal epithelial dysfunction. To date, the common IEC lines used in IBD studies are derived from human colon adenocarcinoma, which are highly differentiated cell lines and have many differences from normal IEC *in vivo* [17]. However, organoid culture technology can simulate the 3D structure and physiological function of organ tissues *in vitro*, and it has become one of the most important biotechnologies in the field of regenerative medicine in the past decade [18]. Intestinal organoids, derived from *Lgr5*^+^ ISCs located mostly at the bottom of the crypt of the intestinal mucosa, can mimic the cell composition and tissue structure of the intestine by recapitulating the self-organizing capacity of cell populations, and it provides a credible model for further investigation of the intestinal barrier in IBD [12, 19]. In this study, we successfully established an intestinal organoid culture system. Consistent with the previous studies, our results showed that Paneth cells and stem cells were located at the budding (crypt-like domain), and fully polarized enterocytes, including goblet cells and enteroendocrine cells, were scattered throughout the villus-like domain. At weekly intervals, the intestinal organoids were mechanically dissociated and replated to one-fifth of the preplating density. A conserved feature of intestinal organoids is that they retain the intestinal lumen where apoptotic enterocytes and metabolites are aggregated. In contrast to the gut epithelium, the growth medium of the intestinal organoids was in contact with the basolateral region (crypt-like region) rather than the enterocyte apical side (villus-like region).

As we know, TNF-*α* is produced by the monocytic lineage and plays an important role in both homeostatic and pathological conditions [20]. Previous studies have shown that patients with chronic intestinal inflammation show elevated TNF-*α* level due to elevated numbers of TNF-*α*-secreting cells in the intestine [21, 22]. Besides immune cells, Paneth cells also constitutively produce TNF-*α* in mice as well as in patients suffering from chronic intestinal inflammation [23]. TNF-*α* is considered a crucial driver of intestinal tissue-damaging inflammatory responses [24]. Mice overexpressing the TNF-*α* gene are more susceptible to chronic TNBS-induced colitis [25]. In this study, among the proinflammatory cytokines IL-1*β*, IL-6, and TNF-*α*, we demonstrated that TNF-*α* could induce the inflammatory response of intestinal organoids, and a dose of 10 ng/mL administered to intestinal organoids could maintain stable passaging (at least 15 passages) of organoids for dynamic observation. The morphology of intestinal organoids mainly has two different states: giant organoids and budding organoids. Intestinal organoids treated with 10 or 40 ng/mL TNF-*α* treatment from day 1 to day 3 were mainly composed of giant organoids. In terms of morphological observations, the apoptotic cells shed into the central lumen with the organoids growing [12, 26]. Our results showed that intestinal organoids with 10 or 40 ng/mL TNF-*α* treatment showed more apoptotic cells in the lumen, and the high concentration of TNF-*α* (dose: 40 ng/mL) treatment hardly maintained continuous growth of intestinal organoids for dynamic observation. So we focused on a more specific dose and time for testing proliferation-related markers *Ki67* and *Lgr5*, and we found that the expressions of *Ki67* and *Lgr5* were decreased significantly after treatment with 10 ng/mL TNF-*α* for 3 days, which suggested that the proliferation ability of intestinal organoids was decreased.

Furthermore, TNF-*α* is a prototypic activator of NF-*κ*B transcription factors and an essential mediator of proinflammatory responses and cell survival [27, 28]. Increased NF-*κ*B expression in mucosal macrophages is accompanied by increased expression of TNF-*α* and chemokines in TNBS- or IL-10-deficient-induced colitis mice [29, 30]. The TNF-*α*/NF-*κ*B pathway participates in the promotion of apoptosis and the inhibition of cell proliferation [31]. The expression of chemokines *Cxcl1*, *Cxcl2*, and *Cxcl10* is dependent on the activation of NF-*κ*B pathway, while the activated inflammatory cytokines and chemokines regulate the inflammation via NF-*κ*B pathway [32]. *IL-15* is a proinflammatory cytokine involved in the development, survival, and proliferation [33, 34]. Our studies found that the expression of inflammatory cytokine *IL-15* and chemokines *Cxcl2* and *Cxcl10* was higher in the intestinal organoids treated with 10 ng/mL TNF-*α*, indicating that TNF-*α* can induce an inflammatory phenotype of intestinal organoids.

Importantly, the normal physiological process of intestinal epithelial shedding was highly regulated by TJP complexes, which rapidly closed the gaps to maintain intestinal barrier integrity [35]. Complex transmembrane proteins interact to form tight junctions [36]. These proteins include occludin and members of the claudin family. The TJPs were also directly linked to cytoskeletal actomyosin fibers via cytoplasmic TJPs, including the zonula occludens (ZO) family [37]. Under physiological conditions, shed cells leave a gap or discontinuity in the epithelium, which is quickly covered by TJPs and filled with new cells generated from crypts. However, tight junction deficiency leads to local permeability and epithelial gaps in inflammatory conditions, especially IBD [38]. Previous studies have reported that high TNF-*α* levels could drive massive epithelial cell shedding at the villus tip and alter tight junctions [39]. Our studies found that the protein expression of occludin and claudin-1 was significantly decreased in intestinal organoids treated with TNF-*α*. However, the mRNA expression level of claudin-1 was not significantly downregulated after treatment with TNF-*α*, and the observed inconsistency may result from posttranscriptional regulation (splicing of hnRNA, RNA editing, RNA interference, etc.), translational regulation, and posttranslational modifications (phosphorylation, glycosylation, ubiquitination, etc.). The comparison of the major TJP (including occludin and claudin-1) changes in our study, other *in vitro* models, and IBD patients is shown in Table 1 [40–47]. The limitations of the current study are the failure to investigate how inflammatory intestinal organoids can restore their ability to proliferate, and the colitis mice are not included as controls, which will be supplemented and establish the intestinal organoids derived from IBD patients in the future study.

## 5. Conclusion

We established the epithelial inflammatory injury model using intestinal organoid cultures that are conducive to the dynamic observation of the growth trajectory and demonstrated that the intestinal organoids with an inflammatory phenotype also contained all cell types of intestinal epithelium. Our study provides a better *in vitro* model of cellular experiments to research the pathogenesis and treatment of IBD.

## Figures and Tables

**Table 1:** Epithelial tight junction changes in intestinal inflammation.

Tight junction		Function	Expression in TNF-*α*-10 treatment	DSS-induced colitis	TNBS-induced colitis	Expression in CD	Expression in UC
mRNA	Ocln	Translated into Ocln protein	↓	↓ [43]	↓ [40]	↓ [41]	↓ [41]
	Cldn1	Translated into Cldn1 protein	↔	↔ [43]	↔ [40]	↓ [41] Active^1^ ↔ [42] Inactive^2^↔ [42]	↓ [41] Active ↔ [42] Inactive ↔ [42]

Protein	Ocln	Binds ZO-1, regulates paracellular permeability, function in cellular adhesion [44]	↓	↓ [43] ↓ [45]	↓ [46]	↓ [47] ↓ [41]	↓ [41]
	Cldn1	Tightens the epithelium able to initiate formation of TJ trends [44]	↓	↓ [43] ↑ [45]	↓ [46]	↓ [41] Active ↔ [47]	↓ [41]

^1^Active represents the sample from the active stage of disease; ^2^inactive represents the sample from the inactive stage of disease. ↓: decrease; ↑: increase; ↔: no change; CD: Crohn's disease; UC: ulcerative colitis; Ocln: occludin; Cldn1: claudin-1.

## Data Availability

The data that support the findings of this study are included within the article and available from the corresponding authors upon reasonable request.

## Supplementary Materials

Supplementary Materials Supplementary Table 1: primer sequences for qRT-PCR.
