# Supplementary material for: Establishment of Epithelial Inflammatory Injury Model Using Intestinal Organoid Cultures
Source: Stem Cells Int. 2023 Mar 7;2023:3328655. doi: 10.1155/2023/3328655 (PMC10014157; doi:10.1155/2023/3328655)
Supplement: Supplementary Materials — Supplementary Table 1: primer sequences for qRT-PCR. [file 3328655.f1.pdf]

Gene name	Gene symbol	Forward primer	Reverse primer
Glyceraldehyde-3-phosphate dehydrogenase	Gapdh	CTGGAAAGCTGTGGCGTGAT	GTCATCATACTTGGCAGGTTTCT
Leucine rich repeat containing G protein coupled receptor 5	Lgr5	CGTTCGTAGGCAACCCTTCT	GGCACCATTCAAAGTCAGTGT
Antigen identified by monoclonal antibody Ki 67	Ki67	CACCACAGAGATTTTGGGAGA	TCTTCAGGGGCTCTGTCTCA
Chemokine (C-X-C motif) ligand 1	Cxcl1	GCAGACCATGGCTGGGATTC	CCGTTACTTGGGGACACCTTTTA
Chemokine (C-X-C motif) ligand 2	Cxcl2	GCTGTCCCTCAACGGAAGAA	GCTGTCCCTCAACGGAAGAA
Chemokine (C-X-C motif) ligand 10	Cxcl10	GTCTGAGTGGGACTCAAGGGAT	TCAACACGTGGGCAGGATAG
Interleukin 15	IL-15	CTCTGCGCCCAAAAGACTTG	GCTGTTAGATGTGGAACTCAGC
Occludin	Ocln	TCCTGGAGGTACTGGTCTCT	TGCAGACACATTTTTAACCCACT
Claudin-1	Cldn1	CAGTTCCGAGGTCTACACCTT	TGAATCGGGAGTCTTCCGAAAA
